# Steeper dose gradients resulting from reduced source to target distance—a planning system independent study

**DOI:** 10.1002/acm2.12490

**Published:** 2018-11-09

**Authors:** Klaus Bratengeier, Kostyantyn Holubyev, Sonja Wegener

**Affiliations:** ^1^ Department of Radiation Oncology University of Würzburg Würzburg Germany; ^2^ Abt. Medizinische Physik University of Freiburg Klinik für Strahlenheilkunde Freiburg Germany

**Keywords:** radiotherapy, stereotactic irradiation, penumbra, leaf width, virtual isocenter

## Abstract

**Purpose:**

To quantify the contribution of penumbra in the improvement of healthy tissue sparing at reduced source‐to‐axis distance (SAD) for simple spherical target and different prescription isodoses (PI).

**Method:**

A TPS‐independent method was used to estimate three‐dimensional (3D) dose distribution for stereotactic treatment of spherical targets of 0.5 cm radius based on single beam two‐dimensional (2D) film dosimetry measurements. 1 cm target constitutes the worst case for the conformation with standard Multi‐Leaf Collimator (MLC) with 0.5 cm leaf width. The measured 2D transverse dose cross‐sections and the profiles in leaf and jaw directions were used to calculate radial dose distribution from isotropic beam arrangement, for both quadratic and circular beam openings, respectively. The results were compared for standard (100 cm) and reduced SAD 70 and 55 cm for different PI.

**Results:**

For practical reduction of SAD using quadratic openings, the improvement of healthy tissue sparing (HTS) at distances up to 3 times the PTV radius was at least 6%–12%; gradient indices (GI) were reduced by 3–39% for PI between 40% and 90%. Except for PI of 80% and 90%, quadratic apertures at SAD 70 cm improved the HTS by up to 20% compared to circular openings at 100 cm or were at least equivalent; GI were 3%–33% lower for reduced SAD in the PI range 40%–70%. For PI = 80% and 90% the results depend on the circular collimator model.

**Conclusion:**

Stereotactic treatments of spherical targets delivered at reduced SAD of 70 or 55 cm using MLC spare healthy tissue around the target at least as good as treatments at SAD 100 cm using circular collimators. The steeper beam penumbra at reduced SAD seems to be as important as perfect target conformity. The authors argue therefore that the beam penumbra width should be addressed in the stereotactic studies.

## 1 INTRODUCTION

Finite Multi‐Leaf Collimator (MLC) leaf width projection is usually believed to limit MLC usage for stereotactic radiotherapy. However, as Bortfeld et al.^1^ showed, it is actually the finite dose deposition kernel and the associated beam penumbra width that in accordance with the sampling theorem make the decrease of MLC leaf width above a certain limit impractical. No physical improvements of the dose distribution are possible at leaf width below 20%–80% penumbra divided by 1.7, which translates into 0.15–0.2 cm leaf widths for 6 MV beams at standard source‐to‐axis distance, with negligible deterioration of dose distribution for doubled leaf width of 0.3 cm. These 0.2–0.3 cm have been accepted rather than confirmed in related publications,^2^, ^3^ and corresponding mini‐ and micro‐MLCs have been developed. To our knowledge, modern studies consider the 0.3 cm leaf width of dedicated STX‐collimators, ignoring the penumbra, except for low energy applications.^4^ Even a recent DEGRO report^5^ refers to the work of Bortfeld et al.,^1^ recommending the leaf width of less than 0.3 cm, but not quantifying necessary penumbra width. On the other hand, studies exist that characterize MLCs for stereotactic purposes; they present measurements of the 20%–80% penumbra,^6^, ^7^, ^8^, ^9^, ^10^, ^11^ however not in a standardized form, that is, they vary in the used depth, beam shape, and beam diameter. In some papers the penumbra is discussed in more detail^12^ or even with respect to its effect on the dose gradient to the healthy tissue.^13^

In the recent planning study for spherical targets,^14^ the authors addressed one possible way to further improve quality of stereotactic plans by using reduced source‐to‐axis distance (SAD). Such reduction can be implemented by placing a target on a robotic couch which co‐moves with the gantry in such a way that the beams cross in the virtual isocenter (VI)^15^ at the “source to virtual isocenter distance” (SVID), closer to the source than the actual machine source to isocenter distance SID, SVID *< *SID, see yendix A. The authors calculated stereotactic plans for different prescription isodoses in Philips Pinnacle^3^™ therapy planning system (TPS) to be delivered at Elekta™ linac equipped with a circular collimator or Agility™ MLC with 0.5 cm leaf width at SID 100 cm, SVID 70 and 55 cm. The target coverage and healthy tissue load at different SVID was compared using van't Riet's/Paddick's conformity index^16^, ^17^ and Paddick's gradient index.^18^ The authors found that Agility™ MLC at SVID 70 cm performs better than circular collimator at standard SID both in terms of target coverage and healthy tissue load. The authors concluded that the improved quality of stereotactic plans at reduced SVID is more due to steeper penumbra than to improved conformity enabled by the reduced MLC leaf width projection.

The purpose of the present work was to verify these results using an independent method which is not based on TPS calculations, to avoid bias from dose calculation algorithms or TPS configuration. Instead, beam profiles in leaf and jaw direction were derived from film measurements at standard and reduced SVID. The isotropic beam arrangement was assumed. An algorithm restricted to spherical targets was developed to transform the two‐dimensional (2D) transverse dose cross‐section of a single beam into three‐dimensional (3D) radial dose distributions from isotropic beam arrangement.

The paper is organized as follows: In the Methods section, the choice of the spherical target and its dimensions are motivated. An algorithm to calculate the mean dose to the spherical shell at a certain radius is derived, using a method similar to that used by Hellerbach et al.^19^ As input, an arbitrary dose distribution of a single beam cross‐section is utilized. The description of the measurement arrangement follows. Finally, parameters necessary to calculate the radial dose distribution for real MLC‐shaped beam and for simulated circular collimators are specified.

In the Results section, the measured beam profiles and the referring calculated radial dose distributions are presented. Comparisons of radial dose distributions and gradient indices of quadratic and circular collimators at several distances and for varying dose prescriptions are performed. We summarize on the potential of standard 0.5 cm leaf MLC combined with SVID to spare healthy tissue around the target, und underline the importance of penumbra characterization in Conclusion.

## 2 METHODS

### 2.A Radial dose distributions from beam cross‐sections

To independently verify the results of a recent planning study^6^, the spherical planning target volume (PTV) of radius R = 0.5 cm was placed at the isocenter or VI, respectively. The realization of VI is described in Appendix A. The target radius R = 0.5 cm presents the worst case for the conformation with even‐numbered 0.5 cm leaf MLC. Also, for SVID the quadratic aperture was maintained, though more conformal shapes would have been available due to the reduced effective leaf width. To distinguish the penumbra‐only effect at SVID from the combined effect of both MLC resolution and penumbra, the dose distributions from circular collimators as perfectly conformal openings were analyzed. As circular collimators were not available at our house, their transverse dose cross‐section was modeled using measured profiles of MLC fields in leaf (L) and jaw (J)—directions as examples for shallow and steep beam edges.

**Figure 1:**
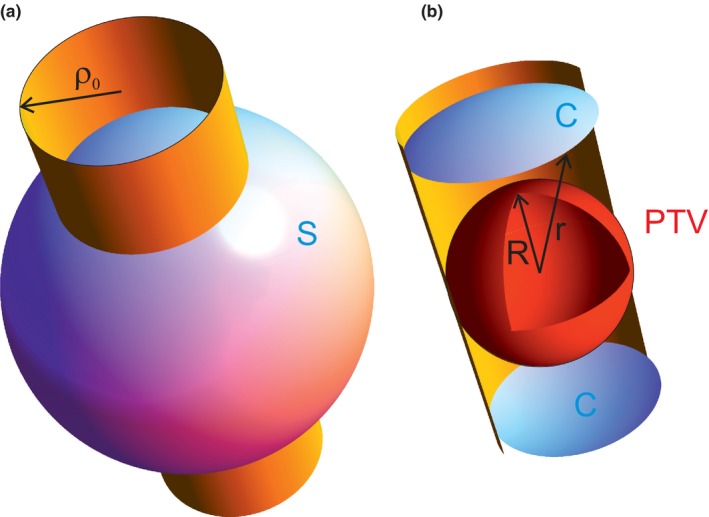
PTV and beam geometry. The spherical PTV of radius *R* (a) is to be irradiated by isotropic beam arrangement. One of the beams with radius *ρ*_*0*_ is shown as a cylinder (b). The dose to the spherical surface S of radius *r* is calculated from the area of the caps *C*.

For spherically symmetric target and isotropic beam arrangement, it is enough to consider only one beam for the dose calculation. The geometry for a circular, PTV adapted top hat beam is shown in Fig. 1. The radial dose *D*(*r*) at *r* is equivalent to the mean dose to infinitely thin spherical shell at radius *r*, Fig. 1(b). For the following, isotropic irradiation and even distribution of collimator angles for non‐circular collimators is assumed. See Appendix C for averaging of collimator rotations for the example of quadratic top hat profiles.

To find the dose *D(r)* from isotropic beam arrangement at distance *r* from the target of radius *R*, we consider a spherical cap *C* cut out by the target‐conformal circular beam of radius *ρ*_0_ = *R* from the surface of radius *r*, Fig. 1(a). If the top hat beam deposits the dose *D*_0_ to the target surface at *r* = *R*, at distance *r* > *R* the same dose is distributed over larger surface, such that the mean dose to a thin shell at *r* is smaller by the ratio of surfaces:

(1) $$D\left(r\right)=d_{0}\frac{2A_{C}\left(r\right)}{A_{0}(\rho_{0)}},$$

where 2*A*_*C*_(*r*) = 4*πrh* is the area of two spherical caps, $h=r-\sqrt{r^{2}-\rho_{0}^{2}}$ is the cap height, and $A_{0}=4\pi \rho_{0}^{2}$ is the area of the sphere at *ρ*_0_. For infinite number of isotropically arranged beams the doses (1) are summed.

**Figure 2:**
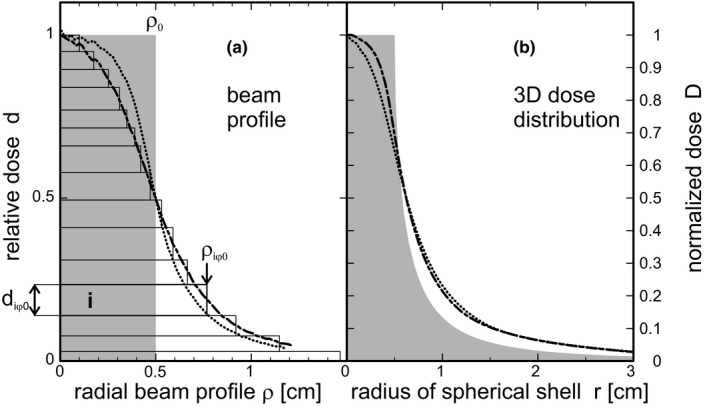
From beam profile to radial dose distribution. (a) The profile of a circular symmetric beam (dot‐dashed) decomposed into a linear superposition of top hat profiles (beam elements *i*). (b) The radial dose distribution is a superposition of the radial dose distributions of top hat profiles. Different profiles result in different radial dose distributions (additional dotted line). Shaded area: PTV. The irregular apertures can be considered a superposition of circular beams of different diameters. The polar angle *φ* is a summation index.

**Figure 3:**
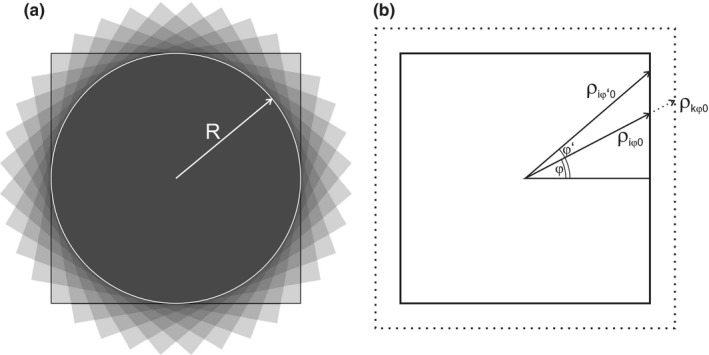
Non‐circular beams. An overlap of many non‐circular beams (shown as squares without loss of generality) behaves like a circular symmetric beam with a modified effective beam profile (a). The azimuthal angle *φ* can be used as additional index to characterize the top hat beam element (b).

Any circular symmetric beam dose profile can be represented as a linear combination of top hat dose profiles, shown as stack of rectangles in Fig. 2(a). An arbitrary non‐circular beam dose cross‐section can be decomposed into a superposition of infinitely thin dose sectors $d_{i\varphi }\left(\rho \right)$, similar to the sector between *φ* and *φ*′ in Fig. 3(b):

(2) $$d\left(\rho \right)=\sum_{i}\sum_{\varphi }d_{i\varphi }\left(\rho \right)$$

with

(3) $$d_{i\varphi }\left(\rho \right)=d_{i\varphi 0}\left[1-H(\rho -\rho_{i\varphi 0})\right],$$

where index *i* runs over the stack of top hat profiles, and *H* is the Heaviside step function. For a circular collimator the dependence on *φ* falls out:

$$d_{i\varphi }\left(\rho \right)=d_{i}\left(\rho \right)$$

It can be easily shown that for a single beam dose component *iφ* with weight *d*_*iφ*0_ and top hat radius of its spherical cap *C*_*iφ*_ equal to *ρ*_*iφ*0_, the contribution to radial dose is

(4) $$D_{i\varphi }\left(r\right)=d_{i\varphi 0}\frac{2A_{C_{i\varphi }}}{A_{0}}=d_{i\varphi 0}\left\{\begin{array}{c} 1\\ \left[1-\sqrt{1-\frac{\rho_{i\varphi 0}^{2}}{r^{2}}}\;\right]\text{for} \end{array}\right.\left\{\begin{array}{c} r\leq \rho_{i\varphi 0}\\ r>\rho_{i\varphi 0} \end{array}\right.$$

The superposition of all dose contributions can be normalized to the dose *D*_0_ at the isocenter or virtual isocenter, respectively:

(5) $$\frac{D(r)}{D_{0}}=\frac{\sum_{i}\sum_{\varphi }D_{i\varphi }\left(r\right)}{\sum_{i}\sum_{\varphi }d_{i\varphi 0}}.$$

Equations (2) and [Link] connect the beam profile $d\left(\rho \right)$ with the radial dose distribution *D*(*r*). Scatter is assumed to be sufficiently considered in the beam profiles as long as they are measured at the target depth. Divergence and absorption are neglected because of the smallness and isocentric position of the target.

### 2.B Film dosimetry

For the calculation of the dose radial distribution according to (2)–(5), the dose transverse cross‐section was measured at gantry position 0° by means of film dosimetry with Gafchromic™ EBT3 film (Ashland, USA). All measurements were performed on an Elekta™ Synergy™ linac with 6 MV photons, flattening filter, and Agility™ head with even‐numbered 0.5 cm leaves. A plastic water slab phantom was used for dose build‐up and backscatter. For the measurement at SID 100 cm, the film was placed at the machine isocenter at a depth of 10 cm, and a nominal 1 × 1 cm² field was irradiated. For the measurement at SVID 70 cm, the film was placed at a distance of 70 cm from the focus, and a nominal field 1.4 × 1.4 cm² (1.0 × 1.0 cm² in the film plane) was irradiated. Similar measurements were performed at the depth of 2 cm and SID 100 and SVID 55 cm, respectively. At SVID 55 cm, the nominal field 2.0 × 2.0 cm² (1.1 × 1.1 cm² in the film plane) was irradiated.

All films were exposed to a maximum dose of approximately 2 Gy. Two reference films were irradiated at 0 and 2 Gy, and evaluated together with the measurements 12 h after exposure following the one‐scan protocol.^20^ All films were centrally placed under a glass slab for compression and scanned at 150 dpi (48 bit RGB) without color corrections on an Epson Expression XL11000 flatbed scanner with a transparency unit. For all cases, the same film orientation and film batch was used. The scans were converted into dose using FilmQA Pro (Ashland, USA) software (see Micke et al.^21^) and digitally stored for evaluation in the lossless.tif format.

To reduce noise, all the profiles were measured twice for SVID 70 cm (single for SVID 55 cm) and symmetrized. The average over four scans resulted in a standard deviation of 0.9% (1.3%, respectively) of the dose maximum. Measured dose distributions were normalized to the mean dose of 0.2 × 0.2 cm² area around the field center.

**Figure 4:**
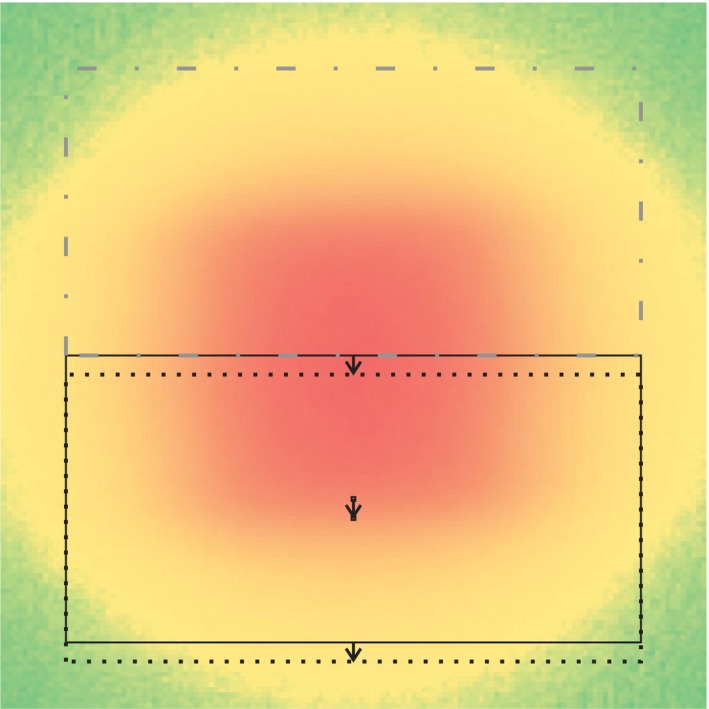
Fine‐tuning of the beam edges. A simple way to adjust the beam edges to get a profile of exactly 0.50 cm half width at half maximum (HWHM) is to shift half‐beams. For overlapping parts only the voxels from one of the halves were used in the overlapping area. If the halves were pulled apart, the voxels in the gap were filled up by interpolation. The shifts have been performed in X and Y direction. For HWHM fine‐tuning necessary shifts were less than 0.04 cm. To achieve a PTV‐surrounding prescription isodose additional shifts could be necessary as noted in Appendix B.

The measured transverse dose cross‐sections were digitally post‐processed to adjust their edges to exactly 1 cm full width at half maximum (FWHM), see Fig. 4. If, after the shift, the halves overlapped, only the voxels from one of the halves were used in the overlapping area. If the halves were pulled apart, the voxels in the gap were filled up by interpolation. For this, the two halves of the beam cross‐section in leaf or jaw direction were shifted by up to 0.04 cm relative to each other.

### 2.C Parameters to be extracted from measurements

For *D*(*r*) calculation according to (5), the contributions for elementary top hat profiles at widths *ρ*_0_ and polar coordinates *φ* have to be summed up. The radial dose profiles $d_{i\varphi }\left(\rho \right)$ were extracted from measured two‐dimensional transverse dose distributions and used to calculate radial dose distribution from isotropic beam arrangement according to (2) and (4), both for quadratic MLC (■) and circular (●) collimators. For quadratic MLC fields (■), the Δ*φ* = 10° sectors were cut from the measured transverse beam cross‐section to use in the calculation.

For the simulation of a circular collimator (●), a linear beam profile was picked out. In the Agility™ head the MLC is sandwiched between the primary collimator and the Y jaws. Thus, the MLC is further away from the target than the Y jaws, which leads to the wider penumbra in the leaf direction, than in the jaw direction. Both types of profiles were used to simulate circular collimator: in the direction of the jaw motion (J) dominated by the jaw penumbra, and in the direction of the leaf motion (L) dominated by the leaf penumbra. The influence of penumbra on healthy tissue load is estimated in Appendix C using a trapezoid beam dose profile as a simplified model for the beam penumbra.

The evaluation of healthy tissue load at different SVID was performed as follows:

1. The ratio of the two distributions was built for direct comparison, and
2. The Paddick's gradient index,^8^ GI, was calculated to facilitate the comparison to the literature.

The GI is the ratio of the volume of half of the prescription dose, V(50%), to prescription isodose volume, V(100%):

$$GI:=\frac{V(50\%)}{V(100\%)}$$

If the prescription isodose line perfectly fits to the target surface, V(100%) is identical to the target volume. Smaller GI values correspond to steeper dose gradient and better healthy tissue sparing.

### 2.D Dose prescription

The calculations according to (2)–(5) were performed for PTV‐surrounding prescription isodoses of 90%, 80%, 70%, and 60% of the isocenter dose, in the following referred to as D90, D80, D70, D60, respectively. In all cases, 100% corresponds to the maximum dose in the isocenter or virtual isocenter.

To ensure a PTV‐surrounding prescription isodose and to enable a direct comparison of radial dose distributions, we digitally shifted the measured cross‐sections to achieve isodose prescriptions D60, D70, D80, or D90. The same method as for the beam width fine‐tuning in section 2B was applied, described in Appendix B and Fig. 4. The necessary shifts were up to 0.06 cm for D60, 0.08 cm for D70, 0.13 cm for D80, and 0.22 cm for D90 prescription.

In order to prove whether any conclusions could be affected by this shifting procedure, additionally GI was calculated without any shifts. It should be noted that the radii used for this GI calculation were not identical with the PTV radius. That is, for D90 and D80, the radius of the prescription isodose was below the PTV radius 0.5 cm, while it is above 0.5 cm for D50 and lower prescription doses. In the following, these prescriptions used for GI calculation were marked with an asterisk, that is, D60*. The related radii were specified.

## 3 RESULTS

**Figure 5:**
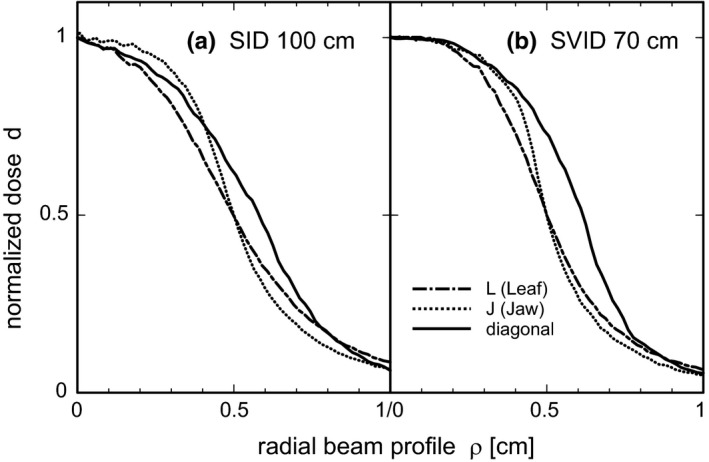
Measured beam edges. Beam edges measured at 10‐cm depth in the solid water phantom by film dosimetry used for calculations of radial dose distribution. Dot‐dashed: leaf direction (L); dotted: jaw direction (J); continuous: diagonals (D) (average over two orientations). (a) Isocentric SID 100 cm (penumbra ∆_20–80_ L 0,44 cm, J 0,31 cm, D 0,40 cm). (b) SVID 70 cm (penumbra ∆_20–80_ L 0,34 cm, J 0,24 cm, D 0,29 cm).

The one‐dimensional beam profiles in leaf and jaw directions, extracted from the measured transverse dose distributions, are shown in Fig. 5 for water‐equivalent depth of 10 cm at SID 100 cm (a) and SVID 70 cm (b). Diagonal profiles are shown for completeness. As expected for Agility™ MLC, the 20%–80% penumbra is wider in leaf direction than in the jaw direction. Finally, the radial dose distribution from isotropic beam arrangement at SID 100 cm and SVID 70 and 55 cm was calculated. For quadratic beam openings, the measured transverse dose cross‐section was digitized. For circular beams the extracted profiles presented in Fig. 5 were used for the calculation of radial dose according to (2). Both profiles in leaf and jaw direction were used to model circular collimators with leaf‐like and jaw‐like penumbra.

**Figure 6:**
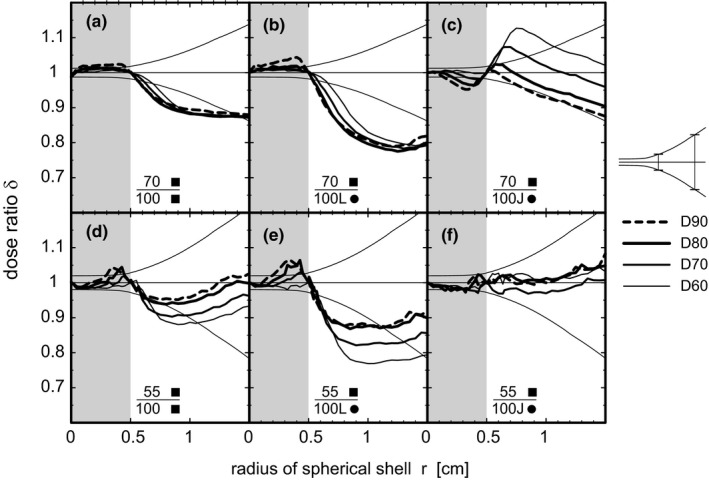
Comparison SVID 70 (55) cm and SID 100 cm. Dose ratio to compare radial dose distributions calculated from beam profiles at different source‐to‐axis distances: “55”: SVID 55 cm, water depth 2 cm; “70”: SVID 70 cm, water depth 10 cm; “100”: SID 100 cm, water depth 2 cm or 10 cm, if compared with “55” and “70”, respectively. “L●”: Circular collimator using leaf‐like edges from film dosimetry (see Fig. 5); “J●”: Circular collimator using jaw‐like edges from film dosimetry (see Fig. 5): “■”: Quadratic collimator from film dosimetry. thin/medium/thick/thick dashed line: prescription to PTV‐surrounding 60%/70%/80%/90% isodose (D60, D70, D80, D90); All doses are normalized to the dose maximum in the PTV center (100%). The light gray area highlights the radii within the PTV. Funnel‐forming fine lines indicate the error bars.

The effect of reduced source‐to‐target distance on healthy tissue load is depicted in Fig. 6 by the dose ratio *δ* of the radial dose distribution at SVID to that at SID. The ratio is shown for SVID 70 cm at depth 10 and 55 cm at 2 cm depth for circular (●), leaf‐like (L), jaw‐like (J), and quadratic (■) collimators and different prescription isodoses. Corresponding GI are presented in Table 1. Here, one should keep the differing definitions of example D60 and D60* from section 2D in mind.

**Table 1:** Gradient indices (GI) for various beam shapes and SID or SVID; R_P_: Prescription radius

Depth	10 cm												2 cm											
SID/SVID [cm]	70		100		70		100		70		100		55		100		55		100		55		100	
Shape, penumbra type	■		■		J●		J●		L●		L●		■		■		J●		J●		L●		L●	
Prescription	R_P_ [cm]	GI	R_P_ [cm]	GI	R_P_ [cm]	GI	R_P_ [cm]	GI	R_P_ [cm]	GI	R_P_ [cm]	GI	R_P_ [cm]	GI	R_P_ [cm]	GI	R_P_ [cm]	GI	R_P_ [cm]	GI	R_P_ [cm]	GI	R_P_ [cm]	GI
D90*	0.33	8.30	0.29	13.64	0.41	4.69	0.36	6.43	0.26	10.88	0.25	19.22	0.39	5.24	0.30	8.51	0.37	4.96	0.30	6.14	0.37	5.66	0.25	14.68
D80*	0.43	4.57	0.42	5.89	0.48	3.37	0.44	4.25	0.35	5.83	0.37	7.91	0.47	3.47	0.38	5.09	0.46	3.02	0.36	4.40	0.46	3.51	0.36	6.26
D70*	0.50	3.62	0.50	4.23	0.53	3.19	0.50	3.65	0.42	4.32	0.45	5.30	0.53	3.03	0.45	3.64	0.50	2.78	0.41	3.56	0.51	3.14	0.44	4.20
D60*	0.56	3.20	0.58	3.55	0.58	3.06	0.55	3.43	0.48	3.81	0.54	4.21	0.58	2.85	0.50	3.27	0.55	2.67	0.46	3.23	0.56	2.96	0.50	3.65
D50*	0.63	2.98	0.66	3.21	0.64	2.95	0.62	3.26	0.54	3.50	0.62	3.63	0.64	2.74	0.57	3.00	0.60	2.75	0.52	3.10	0.62	2.96	0.58	3.15
D45*	0.67	2.91	0.70	3.09	0.68	2.91	0.66	3.18	0.59	3.32	0.67	3.42	0.68	2.72	0.60	2.88	0.62	2.83	0.55	2.98	0.66	2.88	0.62	3.02
D40*	0.72	2.87	0.76	2.99	0.72	2.89	0.71	3.12	0.63	3.26	0.73	3.31	0.72	2.72	0.65	2.81	0.66	2.76	0.59	2.94	0.70	2.79	0.66	2.95
D35*	0.77	2.85	0.81	2.93	0.78	2.81	0.77	3.05	0.68	3.19	0.79	3.17	0.76	2.73	0.69	2.80	0.71	2.74	0.63	2.95	0.75	2.78	0.71	2.91
D30*	0.83	2.83	0.88	2.89	0.85	2.78	0.83	3.02	0.75	3.14	0.87	3.10	0.82	2.78	0.75	2.77	0.76	2.84	0.68	2.84	0.81	2.84	0.77	2.89
D25*	0.91	2.84	0.97	2.92	0.92	2.84	0.92	3.07	0.83	3.07	0.96	3.02	0.90	2.80	0.82	2.78	0.84	2.80	0.75	2.83	0.89	2.88	0.85	2.91

R_P_: Radius for prescription dose.

“L●”: Circular collimator using leaf‐like edges from film dosimetry; “J●“: Circular collimator using jaw‐like edges from film dosimetry; “■”: Quadratic collimator from film dosimetry.

As certain assumptions were made for the dose calculation, the systematic effect of these assumptions needs to be estimated. The absorption has a negligible contribution: The first order Taylor expansion term for absorption at the entrance cap *C* of a beam (Fig. 1) is compensated by the first order term of opposite sign at the exit cap *C*. Even remaining higher order effects compensate, if the ratio of radial dose distributions is considered. The assumption of no divergence contributes as follows. Maximal dose shift due to divergence can be estimated at a distance of about 0.5 cm up to 1 cm from the target center, in the gradient area around PTV. For 1 cm (0.5 cm) the upper limit for deviation is [(SVID+1 cm)/(SID+1 cm)]²/[SVID/SID]², resulting in 1,016 (1,008) for SVID = 55 cm and 1,009 (1,004) for SVID = 70 cm. Due to compensations of the summation at the near and the far cap these dose deviations are even smaller. Phantom scatter is included in the measured beam profiles. Thus, at a conservative estimate, the dose error is dominated by the known error from film dosimetry. Relative errors increase by a factor of $\sqrt{2}$ with respect to the values for the underlying measurements described in section 2B. Funnel‐forming fine lines indicate the error bars in Fig. 6.

The deviation of the ratio from unity, and thus, the improvement of the healthy tissue sparing is only significant at the distances up to 1–2 times the PTV radius. Note, that at least 10% reduction of the healthy tissue dose at SVID = 70 cm compared to SID = 100 cm occurs in this area for all considered dose prescriptions.

**Figure 7:**
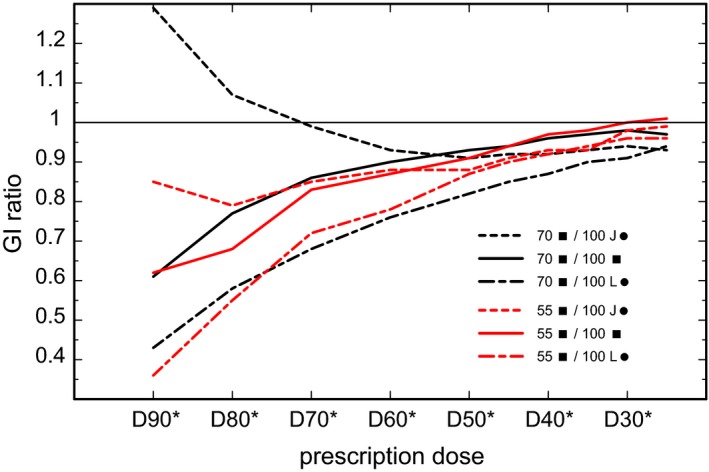
Ratio of gradient indices (GI). Gradient index ratios are depicted for 90%, 80%, 70%, 60%, 50%, 45%, 40%, 35% 30%, and 25% prescription (D25*–D90*), calculated from Table 1. “55”: SVID 55 cm, water depth 2 cm; red or gray lines. “70”: SVID 70 cm, water depth 10 cm; black lines. “100”: SID 100 cm, water depth 2 cm or 10 cm, if compared with “55” and “70”, respectively. “L●”: Circular collimator using leaf‐like edges from film dosimetry (see Fig. 5); “J●”: Circular collimator using jaw‐like edges from film dosimetry (see Fig. 5): “■”: Quadratic collimator from film dosimetry. Continuous line: comparison of quadratic fields, dashed lines: comparison of quadratic field at SVID <100 cm and circular opening at SID 100 cm.

Figure 6(a) and 6(d) show a comparison for quadratic MLC beams, at SVID against SID. The superior healthy tissue sparing of 5%–13% is also reflected by GI reduction as depicted in Fig. 7: A GI ratio of less than 1 appears at a range of prescriptions from D40* to D90*. One should remember that the beam shape in this study remains quadratic at SVID 70 cm, and the MLC conformation can be improved for targets with *R *>* *0.35 cm. Hence, the improved healthy tissue sparing can be attributed directly to the enhanced penumbra at SVID. Without graphical presentation, it should be mentioned that for circular collimators with J‐type and L‐type transverse sections, a sparing between 5% and 20% could be achieved.

The ratios in Fig. 6(b), 6(c), 6(e), and 6(f) refer to the question, whether the penumbra steepening at reduced SVID compensates the non‐conformal quadratic shape, which could be regarded as an extreme form of insufficient MLC beam shaping. For SID and L‐type circular collimators [Fig. 6(b) and 6(e)], the quadratic shape was even better under SVID conditions. For SID and J‐type collimators the sparing was comparable or worse for SVID 70 and D80, D90 [Fig. 6(c) and 6(f)]. These results were underlined by the GI ratio presented in Fig. 7: Only for SVID 70 cm and prescription D80 or D90 the quadratic shape could not compete with the circular collimator at SID 100. In all other cases, the reduced SVID overcompensated the rough, not conformal shaping by the quadratic aperture. The finer resolution by a narrower effective leaf width was not yet taken into account.

Thus, the conclusion of our previous planning study^6^ got an additional confirmation: the penumbra reduction played a leading role in the improvement of healthy tissue sparing at reduced source‐to‐target distance. For the chosen cases in this and the previous study, the penumbra effect was mostly stronger than the effect of a perfect beam shaping by ideally narrow leaves, simulated by the circular collimators.

## 4 DISCUSSION

Bortfeld et al.^1^ discuss the necessary leaf widths as a function of a given beam penumbra width. The penumbra width depends on MLC leaf shape, focal spot size, and primarily on the distance between the patient and the linac. Both parameters, leaf width and penumbra, can be modified. Improvements of one parameter (penumbra) can compensate to a certain degree the disadvantageous “complementary” second parameter (sampling width due to finite leaf size). Therefore, besides the usual leaf width the beam penumbra should equally be quantified in scientific works as well as in recommendations for stereotaxy (STX).

Alternatively, instead of a pair of complementary parameters (penumbra and leaf width), a standard irradiation condition could be specified, that is, the best (lowest) achievable gradient index for the standard stereotactic treatment of a small spherical target; see for example Hellerbach et al^10^ who use spherical targets combined with measured beam characteristics to calculate radial dose distributions and the achievable V12 for the stereotactic treatment of spherical brain metastases with the CyberKnife™ system. The V12 achieved by clinical plans is then benchmarked against this minimal value for the purpose of quality control. In a similar manner, the radial dose distributions can be calculated according to Eqs. (2), (3), [Link], (4) from a measured or theoretical 2D transverse beam dose distribution, and the radiotherapy treatment machine parameters could be integrated into a clinically relevant best achievable reference parameter which can then be used to quantify the quality of clinical plans.^19^ By that, the theoretical limits for gradient indices could be derived from eq. [Link] under assumption of top hat profiles. For example, for the D100 prescription, using eq. [Link], from $D\left(r_{1/2}\right)=\frac{1}{2}D\left(r_{0}\right)$ and $\left(r\right)=1-\sqrt{1-\frac{r_{0}^{2}}{r^{2}}}$, the radius at half of D100 is $r_{1/2}=r_{0}\frac{2}{\sqrt{3}}$, and the best achievable GI for D100 prescription is $GI_{100}=\frac{V\left(D\left(r_{1/2}\right)\right)}{V(D\left(r_{0}\right))}=\frac{8}{\sqrt{27}}\approx 1.54$, which we assume is the theoretical limit. Here, *r*_0_ is the PTV radius and the normalization point at the same time. In comparison, the GI for isocentric and isotropic pencil beams or a point source under assumption of no absorption leads to $GI=\frac{r_{1/2}^{3}}{r_{0}^{3}}=\sqrt{8}\approx 2.83$.

Circular cones dedicated to linac stereotactic radiosurgery (SRS), as found in Ref. 22, 23, 24 vary in their penumbra properties and moreover were characterized under different conditions. Yarahmadi et al.(Fig. 2)^24^ measure in depths d of 5 and 10 cm at source‐surface distance (SSD) 100 cm. Using flatness filter they achieved ∆ = 0.30 cm for a 1 cm cone, which was determined by film dosimetry. With the use of eq. [Link], their beam profile leads to a GI of 3.37 for a D70* prescription (2.84 D70*), which is in the same range as the J● results at SVID 70 cm in this work at d = 10 cm and is slightly better than the MLC ■ results. Borzov et al. [Fig. 4(b)]^22^ describe the properties of a 1 cm cone at SSD 90 cm and d = 10 cm, resulting in ∆ = 0.20 cm with a clearly better D70* GI of 2.75 and a similar D50* GI of 2.79. The beam profile was calculated by a Monte Carlo (MC) model. Morales et al. [Fig. 2(c)]^23^ use a 1 cm cone and measure at SSD 100 cm and d = 10 cm using a micro diamond (1 μm thickness). The stated value of ∆ = 0.15 cm results in D70* GI of 2.43 and a similar D50* GI of 2.54, which is lower than the achievable GI for point sources. Thus, the range of dedicated stereotactic cones is close to and slightly overlapping the range covered in this work by reduced SVID. Further work is needed to explore the opportunities of reduced SVID in more clinical cases. The obtained results have to be compared to irradiations using cones and have to be balanced with the additional need of gantry dependent couch control and quality assurance.

## 5 CONCLUSION

In conclusion, the conventional 0.5 cm leaf MLC that forms a quadratic field and is positioned at SVID of 70 or 55 cm, spares the healthy tissue around a 1 cm spherical target better than the circular collimator at SID of 100 cm, if a leaf‐like penumbra is assumed for the circular collimator. If a jaw‐like penumbra is assumed instead, the healthy tissue sparing of the conventional MLC at SVID and a circular collimator at SID is similar.

As the improvements in the healthy tissue sparing are even underestimated in the considered quadratic geometry, the authors generalize that stereotactic treatments delivered from reduced source‐to‐target distance of 70 or 55 cm using 0.5 cm leaf MLC spare healthy tissue around the target at least as good as treatments from standard source‐to‐target distance of 100 cm using circular collimators. Thus, the houses which practice stereotactic treatments get a flexible solution to deliver stereotactic treatments at reduced source‐to‐target distances with their available linacs and standard MLCs. Furthermore, no special TPS or Record & Verify systems are necessary. Modern TPS should allow all modifications to be implemented as a dedicated virtual linac with SVID < SID. The price for this, however, is the need for a robotic couch to implement a virtual isocenter. The challenges implied by positioning the patient away from machine isocenter should be carefully considered.

Further, the authors have shown that that steeper beam penumbra at reduced source‐to‐axis distance is potentially more important for the planning quality than reduced projected MLC leaf width. Therefore, the beam penumbra width should always be addressed in the stereotactic studies. Moreover, the specification of the beam penumbra should always supplement any technical requirements to the MLC leaf width. Further, as both parameters influence the dose gradient in the vicinity of stereotactic target, the best achievable Paddick's gradient index or similar quantity can be calculated for the standard treatment and used as a reference.

## CONFLICTS OF INTEREST

The authors state that there are no conflicts of interest.

## AUTHORS’ CONTRIBUTION

K. Bratengeier designed the study and performed most of the calculations and was responsible for the theoretical considerations. K. Holubyev discussed the study and was involved in the theoretical considerations. S. Wegener contributed the film dosimetry of real beams.

## Figures and Tables

**Figure 8:**
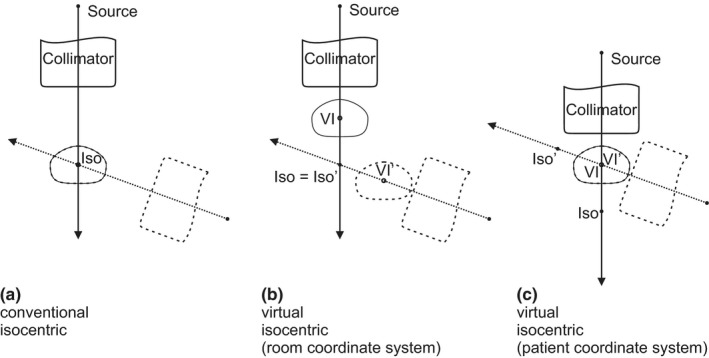
Virtual isocentric against conventional isocentric techniques. Two subsequent gantry positions (dashed lines) for (a) an isocentric technique with conventional source‐to‐axis distance: the beams cross in machine isocenter, at standard SID; (b) a virtual isocentric technique with reduced source‐to‐axis distance; (c) the same technique as seen in the patient coordinate system: the beams cross in the virtual isocenter at SVID < SID. “Iso”: Isocenter.

**Figure C1:**
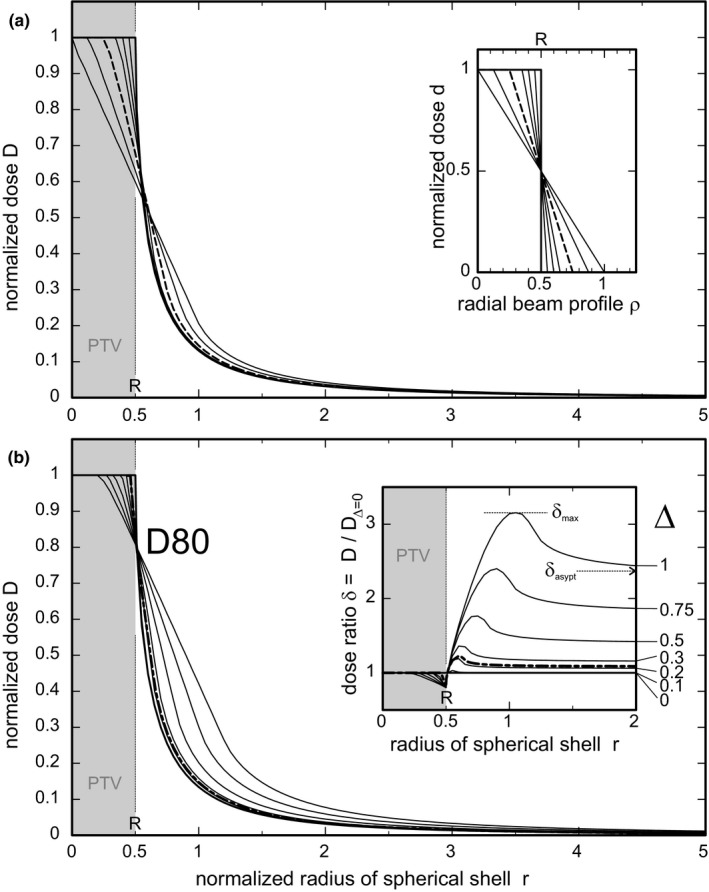
Radial dose distribution for quasi‐isotropic irradiation. Radial dose distribution for quasi‐isotropic irradiation of a spherical target (R = 0.5 cm). (a) Dose distribution for circular symmetric trapezoid beam profiles as depicted in the inset (radial dose). 0%–100% penumbra width Δ = 0, 0.1, 0.2, 0.3, 0.5 (dashed), 0.75, 1.0. (b) Same as above, but shifted beam edge to achieve a PTV‐surrounding isodose of 80% of the maximum dose. Δ = 0, 0.1, 0.2, 0.3, 0.5 0.75, 1.0. Thick line: (ideal) “top hat” circular symmetric beam profile Δ = 0. Dot‐dashed line: quadratic instead of circular beam shape (Δ = 0). Inset: Radial dose ratio of non‐ideal/ideal beam profile.

**Figure C2:**
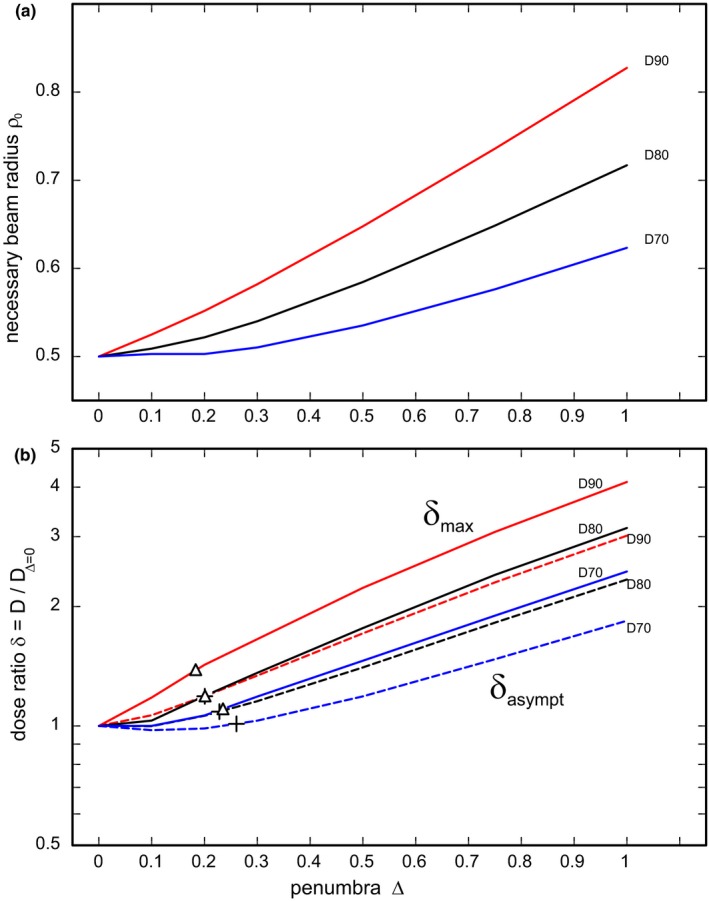
Penumbra effect. (a) Necessary beam width for quasi‐isotropic irradiation of a spherical target (R = 0.5 cm) at 0%–100% penumbra Δ ranging from 0 to 1, for different PTV‐surrounding isodoses of 70% (D70), 80% (D80) and 90% (D90). (b) Resulting maximum dose ratio *δ*_max_ and asymptotic dose ratio *δ*_asympt_ (*r* → ∞) vs 0%–100% penumbra Δ ranging from 0 to 1. Triangles: *δ*_max_ for quadratic instead of circular beam shape for D70, D80 or D90 at the same relative dose than the circular shape assigned to an effective penumbra with the same *δ*_max_. Crosses: similarly, asymptotic dose ratio *δ*_asympt_ for square beams assigned to an effective penumbra.

## APPENDIX A Virtual isocenter (VI)

### A.1

The virtual linac with SVID *<*100 cm can be commissioned in TPS by decreasing SID to SVID, see Fig. A1, and changing the MLC geometry: the field of view and the isocentric leaf width decrease; the dose/MU factor at the calibration point increases according to the inverse‐square law. Obviously, the TPS should allow these modifications at the first hand. Otherwise, the planning process itself does not differ from normal isocentric planning. For a given SVID, the physical couch position can be calculated from gantry angle and couch rotation angle by trigonometric formulas; the realization of the VI should be completely integrated in the couch positioning software. The necessary positioning accuracy is challenging: For example, for SVID 70 cm, the gantry and couch angle have to be set to better than 0.2° to achieve VI positioning to better than 0.1 cm: 0.2° ≈ arctan (0.1 cm/30 cm). Nevertheless, the quality assurance could be performed with the same equipment as for the standard stereotactic techniques (i.e., a Winston‐Lutz^25^, ^26^ test). The further development of the VI concept and the corresponding QA system follows.

## APPENDIX B

### B .1

The shifts to be applied to the halves of the measured transverse dose cross‐sections to obtain necessary PTV‐surrounding isodoses D60, D70, D80, or D90 are noted in tab. B1.

## APPENDIX C

### C.1

To estimate an influence of beam penumbra width on healthy tissue dose in the vicinity of the target, we replaced top head profile (3) in eq. (2) for the trapezoid dose profile^27^ (simplest linear penumbra):

(C1) $$d_{\Delta }\left(\rho \right)=d_{0}\left\{\begin{array}{c} 1\\ \frac{1}{2}-\frac{\rho -\rho_{0}}{\Delta }\;\text{for}\\ 0 \end{array}\right.\left\{\begin{array}{c} \rho \leq \rho_{0}-\frac{\Delta }{2}\\ \rho_{0}-\frac{\Delta }{2}<\rho \leq \rho_{0}+\frac{\Delta }{2}\\ \rho_{0}+\frac{\Delta }{2}<\rho \end{array}\right.$$

$d_{\Delta }\left(\rho \right)$represents a family of trapezoid dose profiles between “top hat” (Δ ≡ Δ_0%–100%_ = 0) and “witch hat” (Δ = 2*ρ*_0_). As can be shown, the corresponding 80%–20% penumbra is Δ_20%–80%_ = 0.6Δ. To estimate penumbra effect, ∆ was varied between 0 cm and 1 cm (∆ = 0, 0.1, 0.2, 0.3, 0.5, 0.75 and 1.0 cm) for spherical target of radius *ρ*_0_ = 0.5 cm. Figure C1(a) shows radial dose distributions from isotropic beam arrangement, calculated according to (2) for circular collimator and trapezoid beam dose profiles *d*_Δ_(*p*) shown in the inset.

Figure C1(b) shows the dose distribution for the circular aperture $d\left(\rho_{0}\right)=0.5d_{0}$ which achieves target coverage with 80% isodose for given penumbra ∆. Here *ρ*_0_ > 0.5 except for ∆ = 0. The inset in Fig. C1(b) shows the ratio *δ* of dose distribution for finite penumbra, ∆ > 0, and vanishing penumbra, ∆ = 0 in the vicinity of the target.

Figure C2(a) shows the radius *ρ*_0_ of circular beam necessary to ensure isodose coverage of the target as a function of penumbra width ∆ for several isodose prescriptions: D70, D80, D90. Obviously, the larger the prescription isodose and the penumbra width, the larger the beam radius *ρ*_0_, necessary to achieve the target coverage. In Fig. C2(b) the maximum of the dose ratio *δ*_*max*_ and its asymptotic value far from the target *δ*_*asympt*_ (see inset in Fig. C1(b) is plotted against penumbra width ∆. Wider penumbra (high ∆) and higher prescription isodose result in higher doses to the healthy tissue around the target.

Furthermore, the effect of square instead of circular beam shapes can be estimated using top hat profiles. For a quadratic shape and rectangular (top hat) dose profile, $d_{i}\left(\rho \right)$ can be calculated analytically:

(C2) $$d\left(\rho \right)=d_{0}\left\{\begin{array}{c} 1\\ 1-\frac{4}{\pi }ArcCos\left(\frac{\rho_{0}}{\rho }\right)\;\text{for}\\ 0 \end{array}\right.\left\{\begin{array}{c} \rho \leq \rho_{0}\\ \rho_{0}<\rho \leq \rho_{0}\sqrt{2}\\ \rho_{0}\sqrt{2}<\rho \end{array}\right.$$

An example of a top hat profile for a square beam has been added in Fig. C1(a) and C1(b), as a dashed line for D80 prescription. Its maximum and asymptotic dose ratios are marked with points in Fig. C2(b). For this square beam, an effective 0%–100% penumbra ∆_*eff*_, leading to the same *δ*_max_ (triangles) and *δ*_asympt_ (crosses) as for the circular beam, was calculated. Values for ∆_*eff*_ ranged from 0.15 to 0.25 and *Δ*_20%–80%,*eff*_ from 0.09 to 0.15. The radial dose distribution of this square beam cannot be distinguished from the dose distribution of a circular beam with 0%–100% penumbra equal to Δ_*eff*_. [see Fig. C1(b)].
